# Diagnosis and Management of Perforated Duodenal Ulcers following Roux-En-Y Gastric Bypass: A Report of Two Cases and a Review of the Literature

**DOI:** 10.1155/2015/353468

**Published:** 2015-04-08

**Authors:** Mazen E. Iskandar, Fiona M. Chory, Elliot R. Goodman, Burton G. Surick

**Affiliations:** Department of Surgery, Mount Sinai Beth Israel Medical Center, New York, NY 10003, USA

## Abstract

Perforated duodenal ulcers are rare complications seen after roux-en-Y gastric bypass (RYGP). They often present as a diagnostic dilemma as they rarely present with pneumoperitoneum on radiologic evaluation. There is no consensus as to the pathophysiology of these ulcers; however expeditious treatment is necessary. We present two patients with perforated duodenal ulcers and a distant history of RYGP who were successfully treated. Their individual surgical management is discussed as well as a literature review. We conclude that, in patients who present with acute abdominal pain and a history of RYGB, perforated ulcer needs to be very high in the differential diagnosis even in the absence of pneumoperitoneum. In these patients an early surgical exploration is paramount to help diagnose and treat these patients.

## 1. Introduction

Peptic ulcer disease and specifically a perforated duodenal ulcer in the excluded stomach or duodenum are a very rare occurrence in patients who have undergone RYGP. Well over one hundred thousand gastric bypass procedures are performed yearly in the USA [1], but only twenty-one cases of perforated duodenal ulcers have been reported in the literature (Table 1) [2–8]. Moreover, most of the reported cases correspond to the early days of gastric bypass when proton pump inhibitors (PPIs) were not as liberally used. The diagnosis of a perforated duodenal ulcer in a RYGP patient can be challenging, and there is variability in the surgical treatment, especially when it comes to the possible role of removing the gastric remnant. We present two cases of a perforated duodenal ulcer following roux-en-Y gastric bypass and discuss the management of these patients.

## 2. Case #1

**Figure 1:**
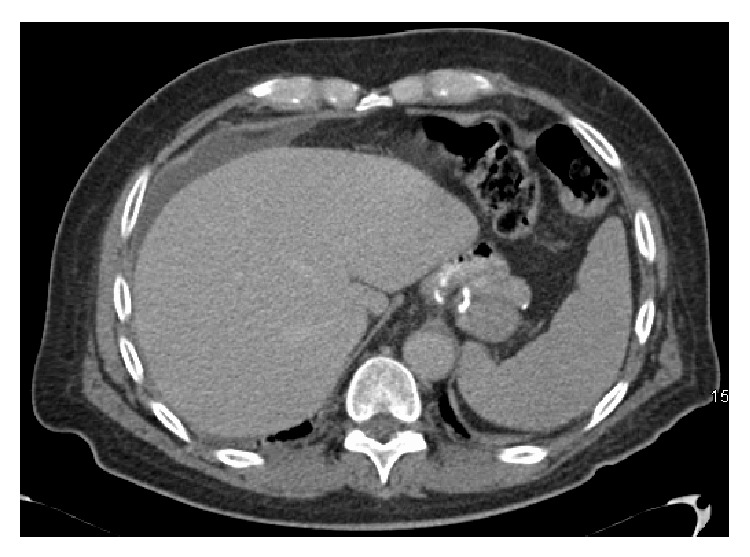
CT showing free fluid in the right paracolic gutter, no free air, and intact gastrojejunal anastomosis.

A 59-year-old male tourist presented to the emergency room with a one-day history of acute onset epigastric pain radiating to the right side of his abdomen and to his back. He denied any other gastrointestinal symptoms and denied taking any nonsteroidal anti-inflammatory agents (NSAIDs). He gave a history of a laparoscopic roux-en-Y gastric bypass performed 10 years priorly at his home country without any short or long term complications. He weighed 125 kilograms before the RYGB (body mass index, BMI 37.7), and he suffered from hypertension and type 2 diabetes mellitus. Following RYGB, his weight nadir was ninety kilograms, and his comorbidities resolved. Physical examination revealed mild tachycardia and tenderness in the epigastrium without evidence of peritonitis. His weight was ninety-six kilograms, and laboratory tests were only significant for an elevated lipase level of 1,043 units/liter (normal range 23–300 units/liter). Chest and abdominal radiographs did not demonstrate free air. A computed tomography (CT) scan with oral and intravenous contrast was obtained that demonstrated a few foci of free air tracking along the falciform ligament, free fluid in the right paracolic gutter, and a distended and thickened gallbladder (Figure 1). There was no extravasation of contrast and the gastrojejunal anastomosis appeared intact. With the concern of a perforated viscus in the excluded segment of the stomach or duodenum, the decision was made for laparoscopic exploration.

Initial exploration revealed bilious ascites that was irrigated and suctioned. Careful inspection of the first portion of the duodenum revealed an 8 mm perforation that was partially sealed by the medial wall of the gallbladder. The defect was closed laparoscopically and primarily using nonabsorbable sutures and buttressed with omentum. Two closed suction drains were left in the subhepatic space next to the duodenum.* Helicobacter pylori* (*H. pylori*) serology was negative. A hepatobiliary iminodiacetic acid (HIDA) scan was obtained postoperatively to make sure that the perforation remained sealed. By the fourth postoperative day, the patient had completely recovered, and the drains were removed. He was seen 1 week after his discharge for a postoperative checkup after which he returned to his country.

## 3. Case #2

A 37-year-old male with history of laparoscopic roux-en-Y gastric bypass in 2002 at an outside institution presented to the emergency department with one week of progressively increasing, sharp epigastric abdominal pain, with a new diffuse quality. It was associated with radiation to the back and development of nausea and emesis in the 24 hours prior to presentation. He denied fever, constipation, obstipation, and NSAID use. His history was significant for peptic ulcer disease and gastrointestinal bleeding from anastomotic erosions. He consumed one bottle of wine daily and had two negative upper endoscopies of his gastric pouch and jejunum, the last one being seven months prior to this admission.

**Figure 2:**
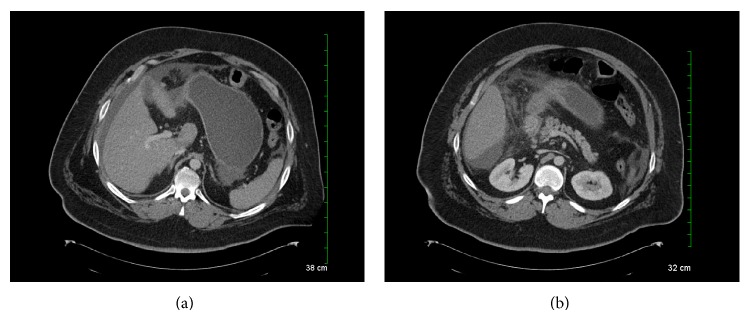
CT demonstrating a distended excluded stomach with perigastric and perihepatic ascites in the absence of pneumoperitoneum and an edematous duodenum with adjacent fat stranding.

On exam he remained morbidly obese BMI 47; he was afebrile and vital signs were within normal limits. He had a soft abdomen with mild epigastric tenderness and no peritoneal signs. His WBC was 9.3 with 79% neutrophils.* H. pylori* serology was negative. Chest and abdominal radiographs did not demonstrate pneumoperitoneum. A computed tomography (CT) scan revealed a markedly distended, fluid-filled excluded stomach and edema of the first portion of the duodenum, jejunum, and transverse colon. There was moderate ascites and no evidence of pneumoperitoneum, and the radiologic diagnosis was enteritis (Figure 2).

**Figure 3:**
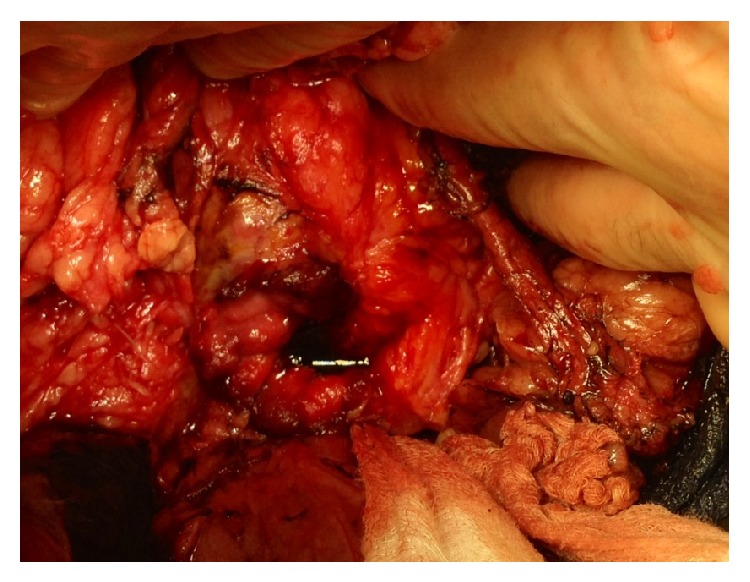
Duodenal defect with bile pooling within defect.

The patient became hypotensive, tachycardic, and diaphoretic and developed worsening abdominal tenderness with guarding shortly after the CT scan. The patient was rapidly optimized in the SICU with fluid resuscitation and vasopressor support and was taken emergently to surgery for an exploratory laparotomy for suspected perforation of the excluded stomach or duodenum. A laparoscopic approach was not considered due to the patient's hemodynamics. At surgery, a large amount of bilious ascites was encountered upon opening the abdomen. The excluded stomach was dilated, and there was a 2-centimeter, 50-percent circumferential duodenal defect in the proximal second portion of the duodenum (Figure 3). The surrounding tissue was very friable and the size of the defect made primary or patch closure impractical. The patient's hemodynamic status was labile intraoperatively and a decision was made to drain the excluded stomach. Two 28 F silicone catheters were placed through the perforation: one was advanced into the excluded stomach and the second into the third portion of the duodenum. The tubes were secured to the edge of the perforation to create a controlled duodenal-cutaneous fistula. A feeding jejunostomy tube was placed, as well as multiple closed suction drains.

By the third postoperative day, the patient was able to be extubated and weaned off vasopressors. His hospitalization was complicated by an upper extremity deep vein thrombosis requiring anticoagulation, acute renal failure which resolved without dialysis, and high output biliary drainage from the silicone catheters. By postoperative day 25 he was discharged tolerating a low fiber diet, on an oral proton-pump inhibitor, anticoagulation, and antibiotics. By the 8th postoperative week, the fistula output was negligible. Subsequently, the tubes were clamped and a HIDA scan revealed preferential flow of bile into the duodenum and none into the fistula. The tubes were removed and the patient has been doing well since.

## 4. Discussion

Diagnosing a perforated duodenal ulcer in a patient following a gastric bypass procedure can be challenging. In a patient who had a prior gastric bypass with acute onset of pain and an acute abdomen, exploration is warranted. However, in a hemodynamically stable patient without peritonitis, imaging provides valuable information in planning operative or nonoperative management. Typically, pneumoperitoneum is absent on radiographs because ingested air would preferentially flow through the gastrojejunostomy rather than retrograde into the biliopancreatic limb. In reviewing the literature there is only one patient where pneumoperitoneum was found on radiologic assessment [3]. In all other patients, the radiographs failed to demonstrate free air. Computed tomography (CT) scan is the most accurate test in making the diagnosing of perforation of the excluded stomach or biliopancreatic limb. The CT scan images will demonstrate free peritoneal fluid, with an inflammatory process in the right upper quadrant. Usually, there will not be any pneumoperitoneum or extravasation of oral contrast. In addition the CT scan will help identify other possible causes of the acute surgical abdomen in a patient after RYGB such as internal herniation (Figure 1).

Several mechanisms have been proposed to explain the pathophysiology of peptic ulcer disease in the excluded stomach and small bowel.* Helicobacter pylori* has been clearly implicated in the formation of ulcers in the gastric bypass population by weakening the mucosal protective barriers [9]. Mucosal injury could also result from the ingestion of nonsteroidal anti-inflammatory drugs (NSAIDs) or excessive alcohol consumption. In the current cases, both* H. Pylori* and NSAIDs were noncontributory but in the second case the consumption of excessive alcohol may have been a factor in the ulcer formation. Bjorkman suggested another mechanism of injury [4]. He postulated that acid produced in the excluded stomach is not neutralized by food as would usually happen in normal anatomy. Moreover, a delay in the release of pancreatic bicarbonate can allow the mucosa to be exposed to the gastric acid for a prolonged period of time. At the same time bile reflux can also damage the mucosa, compounding the effects of the unbuffered acid.

The surgical treatment of perforated duodenal ulcers consists of first the urgent treatment and potentially a more definitive surgical approach. The urgent treatment is usually closure of the defect with an omental patch either through an open or laparoscopic approach. The laparoscopic approach has been shown to be safe in the treatment of perforated marginal ulcers in RYGP patients [10]. A vital question in the treatment of perforated duodenal ulcers in RYGB patients is whether definitive surgery, with completion gastrectomy, is indicated. Resection of the bypassed stomach would lead to a decrease in acid production by eliminating antral gastric secretion. It can also avoid the formation of gastro-gastric fistulae and eliminates the difficult problems of having to access the gastric remnant such as in the case of a bleeding ulcer [8]. However, resection of the excluded stomach is not without consequences and prolongs the operative time. Short term sequelae include duodenal stump leakage and bleeding, and bacterial overgrowth in the biliopancreatic limb and metabolic derangements such as vitamin B 12 deficiency can be seen in the long term [8]. Because of the rarity of this complication and the consequent absence of adequate data, the decision to proceed with a definitive surgical treatment should be based on the particular risks and benefits for each patient. In patients with high operative risk such as case 2, long term PPI therapy is a reasonable alternative.

## 5. Conclusion

Perforated duodenal ulcers following RYGB are rare events and may present a diagnostic challenge as they almost never lead to the formation of free air. Even in the absence of laboratory abnormalities, a high index of suspicion should be maintained, as the presence of free fluid on CT scan may be the only radiologic finding. Surgical exploration remains the mainstay of diagnosis and treatment of acute abdominal pain in RYGB patients. Patients with perforated duodenal ulcers treated with closure in the emergency setting may benefit from resection of the gastric remnant to prevent recurrences but will need to stay on long term PPI therapy.

## Figures and Tables

**Table 1:** Summary of all reported cases with their treatment.

Author/year published	Number of patients	Urgent treatment	Definitive treatment
Moore et al./1979 [2]	2	Closure	Medical
Charuzi et al./1986 [3]	2	Closure	Medical
Bjorkman et al./1989 [4]	1	Medical	Closure/gastrectomy
Macgregor et al./1999 [5]	10	Closure in 9/duodenostomy/gastrostomy in 1	Gastrectomy in 9, medical in 1
Mittermair and Renz/2007 [6]	1	Closure	Medical
Snyder/2007 [7]	4	Closure in 1	Gastrectomy in 3 as initial treatment
Gypen et al./2008 [8]	1	Closure	Gastrectomy
This report	2	Closure in 1/duodenostomy	Medical
